# The Protective Effect of Quercetin against the Cytotoxicity Induced by Fumonisin B1 in Sertoli Cells

**DOI:** 10.3390/ijms25168764

**Published:** 2024-08-12

**Authors:** Jun Ma, Ruixue Huang, Huai Zhang, Dongju Liu, Xiaodong Dong, Yan Xiong, Xianrong Xiong, Daoliang Lan, Wei Fu, Honghong He, Jian Li, Shi Yin

**Affiliations:** 1 Qinghai-Tibetan Plateau Animal Genetic Resource Reservation and Utilization, Southwest Minzu University, Chengdu 610041, China; majun121682@yeah.net (J.M.); huangruixue2022@163.com (R.H.); zhanghuai0224@163.com (H.Z.); qingjiu001020@163.com (D.L.); cehngzi040523@163.com (X.D.); xiongyan0910@126.com (Y.X.); xianrongxiong@163.com (X.X.); landaoliang@163.com (D.L.); fuwei@swun.edu.cn (W.F.); honghong3h@126.com (H.H.); lijian@swun.cn (J.L.); 2 Key Laboratory of Qinghai-Tibetan Plateau Animal Genetic Resource Reservation and Utilization, Ministry of Education, Southwest Minzu University, Chengdu 610041, China; 3 College of Animal & Veterinary Sciences, Southwest Minzu University, Chengdu 610041, China

**Keywords:** fumonisin B1, quercetin, TM4 cell, oxidative stress, glycolysis

## Abstract

Fumonisin B1 (FB1), a mycotoxin produced by *Fusarium* species, is prevalent in crops and animal feed, posing significant health risks to livestock and humans. FB1 induces oxidative stress in Sertoli cells, destroys testicular structure, and affects spermatogenesis. However, methods to mitigate the reproductive toxicity of FB1 in testes remain unknown. Quercetin, a natural flavonoid antioxidant, may offer protective benefits. This study investigated the protective effects and mechanisms of quercetin against FB1-induced reproductive toxicity in TM4 cells (a Sertoli cell line). The results indicated that 40 μM quercetin improved cell viability, reduced apoptosis, and preserved cell functions. Quercetin also decreased reactive oxygen species (ROS) levels in TM4 cells exposed to FB1, enhanced the expression of antioxidant genes, and improved mitochondrial membrane potential. Compared with FB1 alone, the combination of quercetin and FB1 increased ATP levels, as well as pyruvate and lactic acid, the key glycolysis products. Furthermore, this combination elevated the mRNA and protein expression of glycolysis-related genes, including glucose-6-phosphate isomerase 1 (*Gpi1*), hexokinase 2 (*Hk2*), aldolase (*Aldoa*), pyruvate kinase, muscle (*Pkm*), lactate dehydrogenase A (*Ldha*) and phosphofructokinase, liver, B-type (*Pfkl*). Quercetin also boosted the activity of PKM and LDHA, two crucial glycolytic enzymes. In summary, quercetin mitigates FB1-induced toxicity in TM4 cells by reducing ROS levels and enhancing glycolysis. This study offers new insights into preventing and treating FB1-induced toxic damage to the male reproductive system and highlights the potential application of quercetin.

## 1. Introduction

Sertoli cells, the sole somatic cells in direct contact with spermatogenic cells within the seminiferous tubules of the testis, play a crucial regulatory role in spermatogenesis [1,2]. They secrete various factors such as glial cell line-derived neurotrophic factor (GDNF) and platelet-derived growth factor (PDGF), both of which support spermatogenesis [3,4]. Additionally, Sertoli cells form the blood–testis barrier (BTB) via expressing tight junction-related proteins, ensuring a stable micro-environment and immunological barrier for the completion of meiosis [5,6]. They are also vital for maintaining the energy metabolism necessary for spermatogenesis. Lactic acid, a key energy source for germ cells near the spermatogenic tubule lumen, is primarily produced by Sertoli cells [7,8]. Consequently, the regulation of Sertoli cells has garnered increasing attention recently.

Fumonisins are water-soluble secondary metabolites produced by *Fusarium moniliforme*, *F. verticillioides*, and other molds, widely contaminating corn, wheat, sorghum, rice, and other agricultural products globally [9,10]. Fumonisins cause severe oxidative stress damage, posing significant risks to humans and animals. They are linked to esophageal cancer, neural tube defects, and cardiovascular diseases in humans [11,12] and can induce leukomalacia syndrome in horses, pulmonary edema in pigs, and liver and kidney injuries in rodents [13,14]. To date, at least 15 types of fumonisins have been identified, with the B1 type (FB1) being the most toxic and prevalent [15,16]. Recent studies have highlighted FB1-induced reproductive toxicity. For example, FB1 exposure deteriorates oocyte quality by inducing organelle dysfunction, oxidative stress, and DNA damage in mice and pigs [17,18]. Additionally, FB1 affects sex hormone secretion in granulosa cells. After 48 h of treatment with 14 μM FB1, the proliferation of porcine ovarian granulosa cells was inhibited [19]. In mice, FB1 disrupts testicular oxidative balance, leading to inflammation, apoptosis, and testicular tissue damage. In TM4 cells (mouse Sertoli cell line), FB1 increases oxidative stress and apoptosis, demonstrating its toxic effects on Sertoli cells [20]. Lowest observable adverse effect level of 7.50 mg/kg FB1 delayed puberty and impaired semen quality and spermatogenesis in male rabbits [21]. Analysis of fresh and freeze-thawed equine sperm after 60 min of FB1 treatment indicated that 2.5 × 10^−5^ μM FB1 damaged the chromatin structure of frozen samples, while 2.5 × 10^−4^ to 2.5 × 10^−1^ μM FB1 increased reactive oxygen species (ROS) levels [22]. Despite these findings, how to mitigate the reproductive toxicity of FB1 remains largely unknown.

Quercetin is a natural flavonoid with potent antioxidant properties found in various medicinal plants, offering anti-inflammatory, anti-cancer, anti-aging, hypoglycemic, and cardiovascular protective activities [23,24,25,26]. Studies have demonstrated that quercetin can protect Sertoli cells from oxidative stress induced by different chemical agents. For example, quercetin safeguards a Sertoli-germ cell co-culture system from atrazine-induced oxidative stress by reducing ROS levels and lipid peroxidation in rats [27]. Additionally, quercetin mitigates the cytotoxic effects of zinc oxide nanoparticles on TM4 cells by enhancing autophagy and suppressing oxidative stress [28]. This study aimed to determine whether quercetin can alleviate FB1-induced cytotoxicity in Sertoli cells and elucidate the underlying molecular mechanisms. The findings provided new insights into preventing and treating FB1-induced cytotoxicity and the application of quercetin in male reproductive health.

## 2. Results

### 2.1. Quercetin Prevents Cellular Apoptosis and Defective Cell Functions in TM4 Cells Treated with FB1

**Figure 1:**
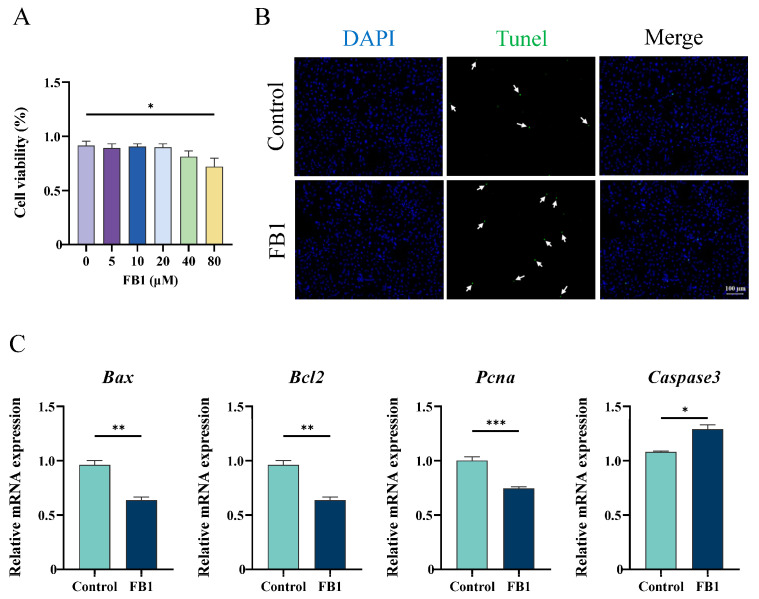
Cytotoxicity detection of FB1 on the viability of the TM4 cell line. (**A**) The CCK-8 assay was performed to detect the proliferation rates of TM4 cells treated with different concentrations (0, 5, 10, 20, 40, and 80 μM) of FB1. * *p* < 0.05, ** *p* < 0.01, *** *p* < 0.001 indicated statistical significance at different levels. One-way ANOVA was employed (with Tukey’s multiple comparison test as the post hoc test). (**B**) Apoptotic cells were detected by TUNEL staining in the Control and 80 μM FB1 group. White arrow indicates the apoptotic cells. Bar: 100 μm. (**C**) Relative mRNA levels of several apoptosis and proliferation-related genes in Control and FB1 group. * *p* < 0.05, ** *p* < 0.01, *** *p* < 0.001 indicated statistical significance at different levels; Student’s unpaired *t*-test was employed. Data presented in (**A**,**C**) are expressed as mean ± SEM from three independent experiments.

Cell viability in TM4 cells treated with FB1 of different concentrations (0, 5, 10, 20, 40, and 80 μM) was assessed using the Cell Counting Kit-8 (CCK-8) assay. Compared with the Control group (the cells were not treated with any drugs), cell viability significantly decreased by 29.4% in the 80 μM FB1 treatment group (FB1 group) (Figure 1A). TUNEL assay results further confirmed increased apoptosis in the 80 μM FB1 group (Figure 1B). Additionally, mRNA expressions of apoptosis and proliferation-related genes, including Bcl2-associated X protein (Bax), B cell leukemia/lymphoma 2 (Bcl2), caspase3 (Casp3), and proliferating cell nuclear antigen (Pcna), were detected. FB1 treatment suppressed the expression of the anti-apoptotic gene Bcl2 and the proliferation-related gene Pcna while significantly increasing the expression of the pro-apoptotic gene Bax (Figure 1C). These findings confirm that 80 μM FB1 induces apoptosis in TM4 cells.

**Figure 2:**
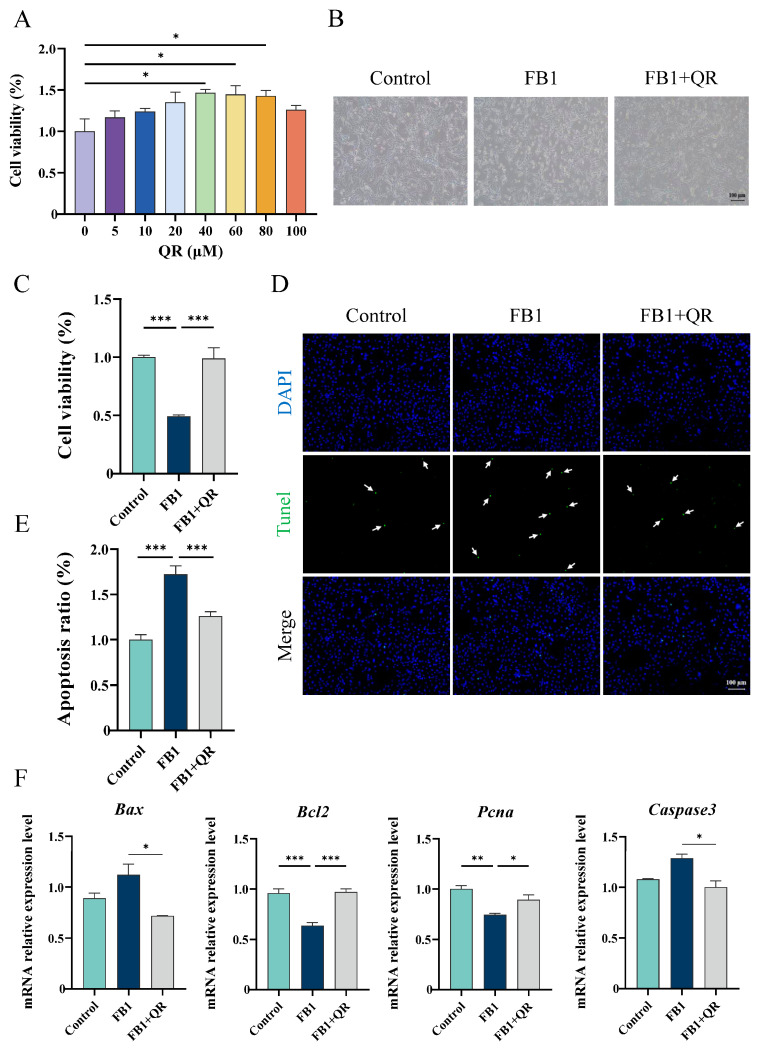
Effect of quercetin on the viability of TM4 cell lines treated with fumonisin B1 (FB1). (**A**) The CCK-8 assay was performed to detect the proliferation rates of TM4 cells treated with different concentrations (0, 5, 10, 20, 40, 60, 80, and 100 μM) of quercetin. (**B**) Representative images of cell morphology in Control, 80 μM FB1 (FB1) and 80 μM FB1 + 40 μM quercetin (FB1 + QR) groups. Bar: 100 μm. (**C**) Cell viability in Control, FB1, and FB1 + QR groups. (**D**) Apoptotic cells detected by TUNEL staining in Control, FB1, and FB1 + QR groups. White arrows indicate the apoptotic cells. Bar: 100 μm. (**E**) Apoptosis ratio analysis in (**D**). (**F**) Relative mRNA levels of several proliferation and apoptosis-related genes in Control, FB1, and FB1 + QR groups. * *p* < 0.05, ** *p* < 0.01, *** *p* < 0.001 indicated statistical significance at different levels; one-way analysis of variance (ANOVA) was employed (with Tukey’s multiple comparisons test as the post hoc test). Data presented in (**A**,**C**,**E**,**F**) are expressed as mean ± standard error of the mean (SEM) from three independent experiments.

To examine the protective effect of quercetin on FB1-treated TM4 cells, cell viability was measured using the CCK-8 assay with quercetin of different concentrations (0, 5, 10, 20, 40, 60, 80, and 100 μM). Cell viability initially increased with higher quercetin concentrations, peaking at 40 μM, before declining (Figure 2A). Further analysis indicated that co-treatment with 40 μM quercetin and 80 μM FB1 (FB1 + QR group) significantly rescued cell viability compared with the FB1 group alone (Figure 2B,C) and reduced apoptosis (Figure 2D,E). Additionally, the expressions of Pcna and Bcl2 increased by 20.2% and 52.4%, respectively, in the FB1 + QR group compared with FB1 group, while the pro-apoptotic genes Bax and Caspase3 were significantly down-regulated (Figure 2F).

**Figure 3:**
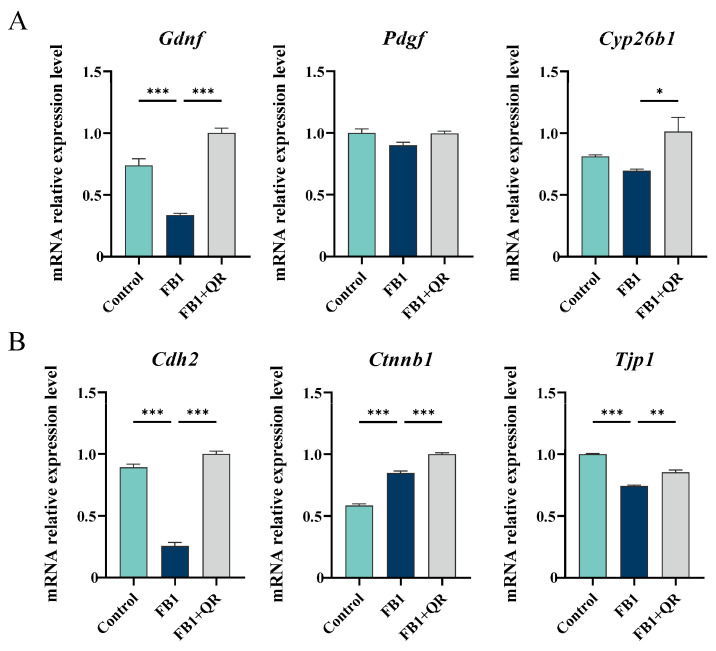
The impact of quercetin treatment on mRNA expressions of Sertoli cell function-related genes in FB1-treated TM4 cells. (**A**) Relative mRNA levels of several Sertoli cell secretory factors responsible for spermatogenesis in Control, FB1, and FB1 + QR groups. (**B**) Relative mRNA levels of several genes encoding BTB-related proteins in Control, FB1, and FB1 + QR groups. * *p* < 0.05, ** *p* < 0.01, *** *p* < 0.001 indicated statistical significance at different levels; one-way ANOVA was employed (with Tukey’s multiple comparisons test as the post hoc test). Data presented are expressed as mean ± SEM from three independent experiments.

Given that Sertoli cells support spermatogenesis through paracrine signaling and by forming the BTB, the mRNA expressions of several spermatogenesis-related factors secreted by Sertoli cells, including Gdnf, Pdgf, and cytochrome P450, family 26, subfamily b, and polypeptide 1 (Cyp26b1), were detected. The expressions of Gdnf and Cyp26b1 were significantly up-regulated in the FB1 + QR group compared with the FB1 group. The mRNA levels of BTB components, including cadherin 2 (Cdh2), catenin beta 1 (Ctnnb1) and tight junction protein 1 (Tjp1), were detected and both Cdh2 and Tjp1 were suppressed in the FB1 group but restored with the combined FB1 and quercetin treatment (Figure 3).

### 2.2. Quercetin Eliminates Excess Oxidative Stress and Restores the Mitochondrial Membrane Potential Damage in TM4 Cells Treated with FB1

**Figure 4:**
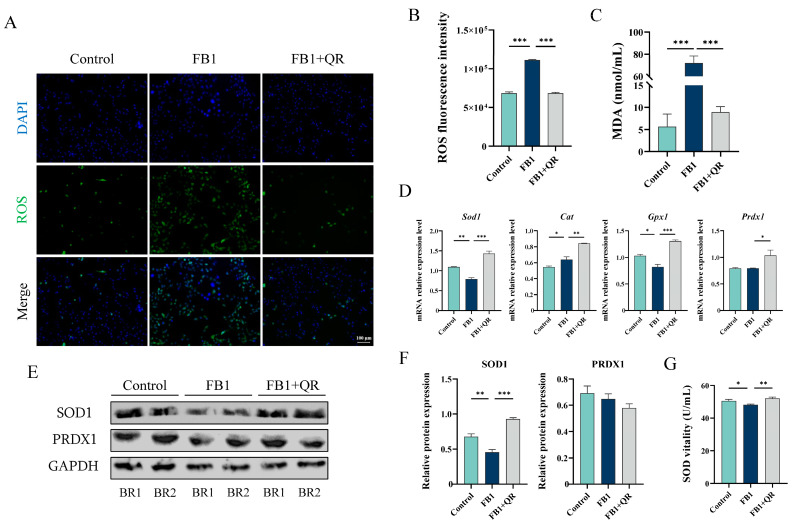
Quercetin attenuates FB1-induced oxidative damage in the TM4 cell line. (**A**) Detection of ROS levels in Control, FB1, and FB1 + QR groups by treating cells with DCFH-DA diacetate. Bar: 100 μm. (**B**) Reactive oxygen species (ROS) levels in (**A**) were quantified by measuring the fluorescence intensity of DCFH-DA. (**C**) Detection of malondialdehyde (MDA) levels in Control, FB1, and FB1 + QR groups. (**D**) Relative mRNA levels of several antioxidant genes in Control, FB1, and FB1 + QR groups. (**E**) Protein expressions of antioxidant enzymes superoxide dismutase 1 (SOD1) and peroxiredoxin 1 (PRDX1) in Control, FB1, and FB1 + QR groups. BR1 and BR2 represents two independent biological replicates, the same as below. (**F**) Optical density analysis of protein expressions of SOD1 and PRDX1 in (**E**). (**G**) SOD activity detection in Control, FB1, and FB1 + QR groups. * *p* < 0.05, ** *p* < 0.01, *** *p* < 0.001 indicated statistical significance at different levels; one-way ANOVA was employed (with Tukey’s multiple comparisons test as the post hoc test). Data presented in (**B**–**E**,**G**) are expressed as mean ± SEM from three independent experiments.

Considering that FB1 induces excess oxidative stress in Sertoli cells and quercetin is a potent antioxidant, we evaluated ROS levels in TM4 cells after combined FB1 and quercetin treatment. Compared with the FB1 group, ROS levels decreased by 38.5% in the FB1 + QR group (Figure 4A,B). Malondialdehyde (MDA), an indicator of lipid oxidation and oxidative stress, significantly increased in the FB1 group compared with the Control group but decreased in the FB1 + QR group (Figure 4C).

The expressions of several antioxidant genes, including superoxide dismutase 1 (Sod1), catalase (Cat), glutathione peroxidase 1 (Gpx1) and peroxiredoxin 1 (Prdx1), were also measured. In the FB1 + QR group, the expressions of Sod1, Gpx1, and Prdx1 increased by 81.5%, 59.3%, and 30.9%, respectively, compared with the FB1 group (Figure 4D). Similarly, the protein expression of SOD displayed similar expression tendency (Figure 4E,F). The activity of SOD also significantly increased in the FB1 + QR group compared with that of the FB1 group (Figure 4G).

**Figure 5:**
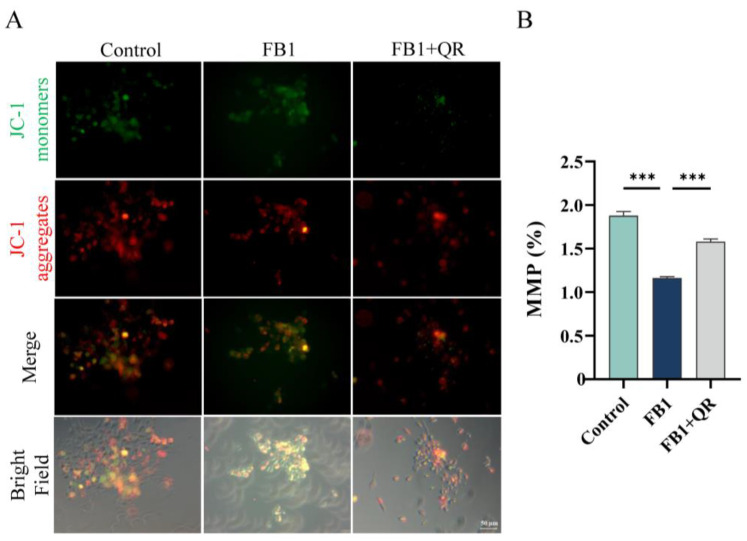
Quercetin restores mitochondrial membrane potential (MMP) in TM4 cells treated by FB1**.** (**A**) Mitochondrial membrane potential was detected by JC-1 fluorescent probe in Control, FB1, and FB1 + QR groups. JC1-monomers and JC1-aggregates produce green and red fluorescence, respectively. Bar: 50 μm. (**B**) Mitochondrial membrane potential (MMP) in (**A**) was quantified by measuring the ratio of fluorescence intensity of JC1-aggregates to JC1-monomers. *** *p* < 0.001 indicated statistical significance at different levels; one-way ANOVA was employed (with Tukey’s multiple comparisons test as the post hoc test). Data presented in (**B**) are expressed as mean ± SEM from three independent experiments.

Given that mitochondria are primary targets of FB1 and mitochondrial dysfunction is a secondary effect responsible for FB1-induced ROS generation [29], we examined the distribution and membrane potential of mitochondria (MMP) in TM4 cells under different treatments. Mito-Tracker probing revealed no significant differences in mitochondrial distribution (Figure 1). However, FB1 treatment significantly decreased MMP by 38.1% compared with that of the Control group, while the FB1 + QR group exhibited a 35.8% increase in MMP compared with the FB1 group, indicating that quercetin ameliorated FB1-induced mitochondrial membrane potential damage (Figure 5).

### 2.3. Dynamic Transcriptome Changes in TM4 Cells Treated with FB1 and Quercetin

**Figure 6:**
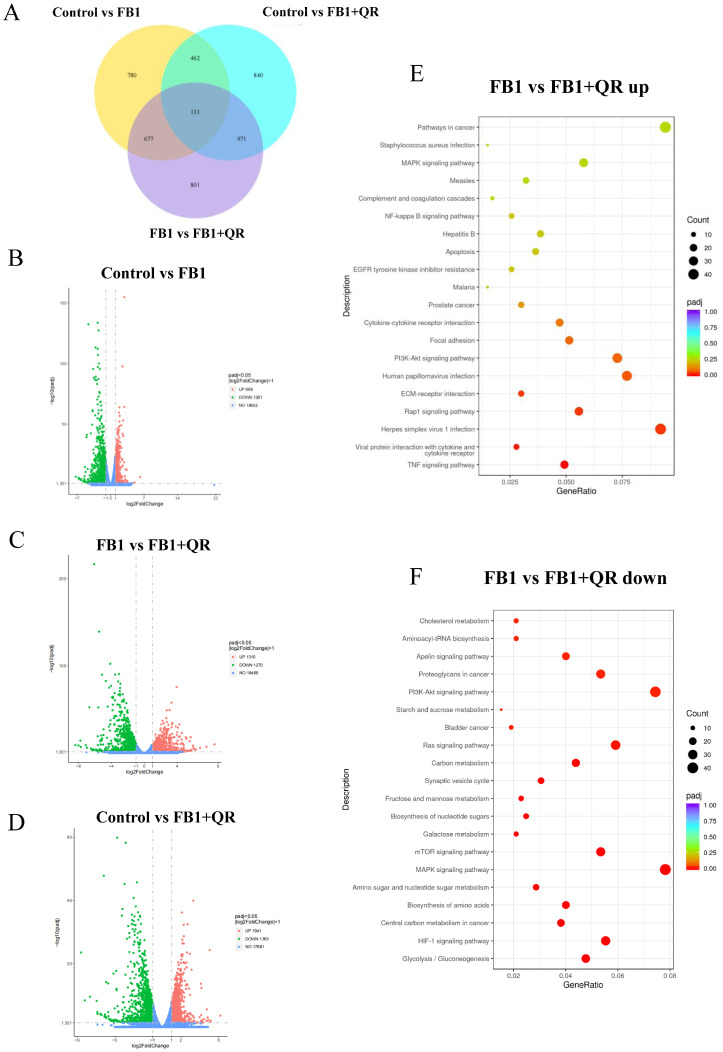
Transcriptome analysis of TM4 cell line treated with FB1 and quercetin. (**A**) Venn Diagram of differentially expressed genes (DEGs) in Control versus (vs.) FB1, Control vs. FB1 + QR, and FB1 vs. FB1 + QR groups. (**B**–**D**) Volcano plot of all transcripts in Control vs. FB1 (**B**), Control vs. FB1 + QR (**C**), and FB1 vs. FB1 + QR groups (**D**). The Y-axis represents the negative logarithm value of the error detection rate. Blue, red, and green dots represent genes with no significant change, significantly up-regulated genes, and significantly down-regulated genes. (**E**) KEGG analysis of down-regulated gene enriched processes in FB1 vs. FB1 + QR groups. (**F**) KEGG analysis of up-regulated gene enriched processes in FB1 vs. FB1 + QR groups.

To further explore the mechanism by which quercetin protects against FB1-induced cytotoxicity in TM4 cells, mRNA from the Control, FB1, and FB1 + QR groups was collected and sequenced. A total of 2050, 2404, and 2580 differentially expressed (DE) transcripts were identified in the Control versus (vs.) FB1, Control vs. FB1 + QR, and FB1 vs. FB1 + QR groups, respectively (Figure 6A–D, Tables S2–S4). The accuracy of the sequencing data was confirmed by real-time PCR (Figure S2). KEGG enrichment analysis was then applied to identify the altered biological processes. In the FB1 vs. FB1 + QR group, the significantly down-regulated genes in the FB1 vs. FB1 + QR group were enriched in pathways such as Glycolysis/Gluconeogenesis, HIF-1 signaling pathway, and Central carbon metabolism in cancer (mmu05230) (Figure 6E, Table S5). Meanwhile, the significantly up-regulated genes in the FB1 vs. FB1 + QR group were enriched in pathways including the TNF signaling pathway (mmu04668), Viral protein interaction with cytokine and cytokine receptor (mmu04061), and Herpes simplex virus 1 infection (mmu05168) (Figure 6F, Table S6).

### 2.4. Quercetin Treatment Restored the Defective Energy Metabolism Level in FB1-Treated TM4 Cells 

**Figure 7:**
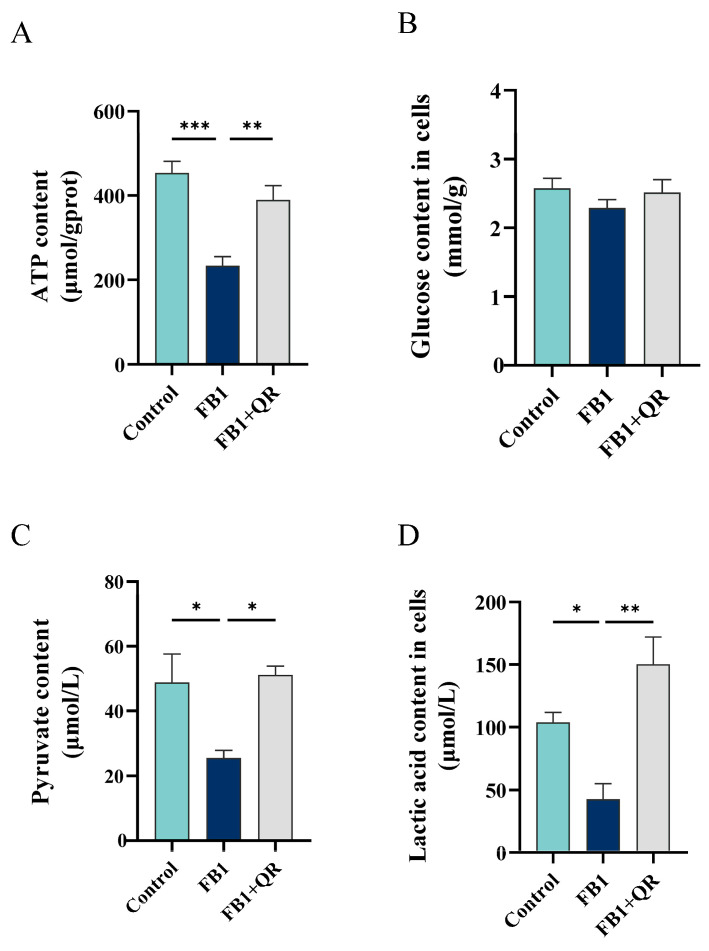
Quercetin enhances glycolysis in FB1-treated TM4 Cells. (**A**–**D**) Intracellular contents of (**A**) ATP, (**B**) glucose, (**C**) pyruvate, (**D**) and lactic acid. * *p* < 0.05, ** *p* < 0.01, *** *p* < 0.001 indicated statistical significance at different levels; one-way ANOVA was employed (with Tukey’s multiple comparisons test as the post hoc test). Data presented are expressed as mean ± SEM from three independent experiments.

Glycolysis is crucial for Sertoli cells to obtain energy. To determine if the glycolysis process is affected in TM4 cells treated with FB1 and quercetin, we first measured ATP production. FB1 treatment resulted in a 48.5% decrease in ATP levels compared to the Control group, while the addition of quercetin increased ATP levels by 66.8% (Figure 7A). We then examined the contents of glucose, pyruvate, and lactic acid, key metabolites in glycolysis, in TM4 cells under different treatments. Although glucose levels indicated no significant differences among the groups, both pyruvate and lactic acid levels significantly decreased in the FB1 group compared with the Control group and were restored in the FB1 + QR group (Figure 7B–D). This indicated that FB1 disrupts the glycolysis process in TM4 cells, and quercetin treatment restores energy metabolism.

**Figure 8:**
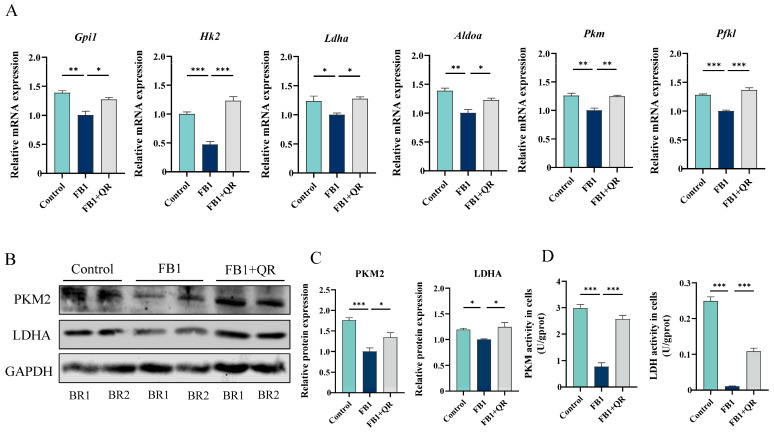
Quercetin regulates the expression and activity of glycolysis-related enzymes. (**A**) Relative mRNA levels of several antioxidant glycolysis-related genes in Control, FB1, and FB1 + QR groups. (**B**) Relative protein expression of glycolysis-related proteins PKM2 and LDHA in Control, FB1, and FB1 + QR groups. (**C**) Optical density analysis of protein expressions of PKM2 and LDHA in (**B**). (**D**) Activities of PKM and LDHA in different groups. * *p* < 0.05, ** *p* < 0.01, *** *p* < 0.001 indicated statistical significance at different levels; one-way ANOVA was employed (with Tukey’s multiple comparisons test as the post hoc test). Data presented are expressed as mean ± SEM from three independent experiments.

RNA-seq results revealed that six glycolysis-related genes, including hexokinase 2 (Hk2), glucose-6-phosphate isomerase 1 (Gpi1), aldolase (Aldoa), phosphofructokinase, liver, B-type (Pfkl), pyruvate kinase, muscle (Pkm), and lactate dehydrogenase A (Ldha) were significantly up-regulated in the FB1 + QR group compared with the FB1 group (Figure S2). Real-time PCR confirmed that all of these genes were significantly down-regulated in the Control vs. FB1 group and up-regulated in the FB1 vs. FB1 + QR group (Figure 8A). PKM and LDHA are key enzymes in the glycolytic pathway, converting phosphoenolpyruvate and ADP into pyruvate and ATP, and catalyzing the conversion between lactic acid and pyruvate, respectively. Protein expression of PKM2, the isomer of PKM mainly expressed in testicular Sertoli cells, decreased in the Control vs. FB1 group and increased in the FB1 vs. FB1 + QR group. The protein expression of LDHA showed a similar trend (Figure 8B,C). The activities of both enzymes were repressed in FB1-treated cells and restored after combined treatment with FB1 and quercetin (Figure 8D). This repression suggested that quercetin may restore defective energy metabolism in FB1-treated TM4 cells by up-regulating the expression and activity of glycolysis-related enzymes.

## 3. Discussion

Fumonisin pollution is widespread in corn, wheat, sorghum, rice, and other food crops, posing a significant threat to human and livestock health throughout the food chain. Among various fumonisins, FB1 is the most toxic and prevalent [30,31]. While several studies have confirmed the toxic effects of FB1 on reproductive organ cells, such as oocytes, granulosa cells, and Sertoli cells [18,19,20,32,33], little is known about mitigating its reproductive toxicity. This study is the first to demonstrate that quercetin, a natural flavonoid, can reduce FB1-induced toxicity in TM4 cell lines by lowering oxidative stress levels and enhancing energy metabolism.

We demonstrated that the toxic effect of FB1 on TM4 cells increases with its concentration, ranging from 0 to 80 μM. Treatment with 80 μM FB1 significantly increased the number of apoptotic cells and oxidative stress levels in TM4 cells compared with the Control group, with notable up-regulation of pro-apoptotic genes *Caspase3* and *Bax* (Figure 1). These findings are consistent with the toxic effects of FB1 on other cells, such as pig small intestine epithelial cells and human kidney tubular epithelial cells [34,35]. It has been reported that quercetin has a scavenging effect on ROS production induced by chemical factors or heat stress in Sertoli cells [28,36]. Here we found quercetin supplementation reduced intracellular oxidative stress, increased the expression of several antioxidant genes, and enhanced both the expression and activity of *Sod*, thereby improving FB1-treated TM4 cell viability. These results suggested that quercetin had a conservative effect on ROS clearance induced by different conditions. A decrease in mitochondrial membrane potential is a known adverse effect of oxidative stress imbalance and initiates a series of biological changes leading to apoptosis [37,38]. We also observed that FB1 treatment significantly reduced mitochondrial membrane potential, while quercetin could reverse this change. Together, these results indicate that quercetin protects TM4 cells from FB1-induced cytotoxicity by enhancing antioxidant capacity and alleviating oxidative stress.

Sertoli cells exhibit metabolic processes that bear resemblance to those of tumor cells. Research indicates that the impact of quercetin on tumor cell survival may be dependent on the dosage of the drug and the duration of treatment. For instance, the viability of hydrogen peroxide-treated K562 cells was observed to increase with a low dose of quercetin (5–100 μM) over a 3-h period, whereas higher doses of quercetin (100–500 μM, 24 h) were found to be toxic, leading to a decrease in cell viability [39]. One potential explanation is that at lower concentrations, quercetin functions as an antioxidant, exerting chemopreventive effects. Conversely, at higher concentrations, quercetin acts as a pro-oxidant, triggering apoptosis, cell cycle arrest, and inhibition of cell proliferation [40]. Our findings suggest that treating Sertoli cells with quercetin within the range of 0–80 μM for 48 h can enhance cell viability. However, the impact of higher doses and longer exposure periods of quercetin on Sertoli cell viability warrants further investigation.

The role of the BTB is to isolate germ cells located in the adluminal compartment from the circulatory system. Together with local immune suppression, it forms an immunological barrier that prevents the passage of antigens present in postmeiotic germ cells during spermiogenesis. The BTB is composed of tight junctions, ectoplasmic specializations, desmosomes, and gap junctions located between Sertoli cells [6]. Various studies have demonstrated that FB1 influences the expression of proteins related to the BTB. For instance, FB1 has been shown to decrease the expression of genes associated with tight junctions such as *CLAUDIN 1*, *OCCLUDIN*, and *TJP1* in swine umbilical vein endothelial cells and intestinal epithelial cells [41,42]. In this study, we observed that FB1 suppressed the expression of BTB-related genes *Cdh2* and *Tjp1*, but their levels were restored following treatment with quercetin. CDH2 is a protein related to basal ectoplasmic specializations and is involved in intercellular Ca^2+^-dependent adhesion [43]. TJP1 is an adaptor protein that facilitates the attachment of integral membrane proteins of tight junctions, such as Occludin and Claudin, to the actin cytoskeleton [6]. Our findings suggest that FB1 may disrupt the structure of tight junctions and ectoplasmic specializations, while quercetin potentially reverses this effect. Further in vivo experiments are necessary to validate this hypothesis.

Energy metabolism is crucial for cell viability, with glycolysis being the initial stage. Sertoli cells can perform glycolysis under aerobic conditions, producing ATP and various metabolites, a phenomenon known as the “Warburg effect” [8,44]. Transcriptome sequencing indicated that glycolysis might be affected by FB1 and quercetin treatments. Pyruvate, a key glycolytic intermediate, can be reduced to lactic acid in the cytoplasm for energy or participate in the intracellular conversion of sugars, fats, and amino acids [45]. Our results indicated significant decreases in ATP and pyruvate levels in the FB1 vs. Control group, which were significantly restored in the FB1 + QR vs. FB1 group. Although the intracellular glucose content did not change significantly after FB1 and quercetin treatments, the expression of several genes encoding enzymes that convert glucose to pyruvate, including *Gpi1*, *Hk2*, *Ldha*, *Aldoa*, *Pfkl* and *Pkm*, were significantly up-regulated in the FB1 + QR group compared to the FB1 group. PKM2, a rate-limiting enzyme in glycolysis responsible for generating pyruvate and ATP from phosphoenolpyruvate and ADP in Sertoli cells [46,47,48], was repressed in both expression and activity by FB1 treatment. The addition of quercetin reversed this repression, suggesting that PKM may be a key target of quercetin in alleviating FB1-induced cytotoxicity in TM4 cells.

Lactic acid, the end product of glycolysis, serves dual roles: maintaining normal energy metabolism and survival of Sertoli cells and providing an energy source for spermatogenic cells [4,49]. Our measurements of intracellular lactate content suggested that FB1 inhibits lactate synthesis in TM4 cells, while quercetin relieves this inhibition. Our results proved that both the expression and activity of LDH, the enzyme catalyzing the conversion between pyruvate and lactic acid [50,51], was significantly inhibited by FB1 and restored by quercetin treatment. Since pyruvate is the main substrate for lactate synthesis, the observed changes in lactic acid content are likely due to alterations in pyruvate content, LDH expression and its activity. 

Our results demonstrated that quercetin positively regulates glycolysis in FB1-treated TM4 cells. Interestingly, quercetin has been reported to inhibit glycolysis in certain tumor cells, such as oral squamous cell carcinoma and breast cancer [52,53]. Given that tumor cell metabolism is highly vigorous and the glycolysis in FB1-treated TM4 cells is inhibited, we speculated that the regulatory effect of quercetin on glycolysis may depend on the metabolic state of the cell. Beyond sugar metabolism, transcriptome sequencing data revealed that several Sertoli cell-related pathways, including the TNF signaling pathway, Ras signaling pathway, and mTOR signaling pathway, are altered in TM4 cells treated with FB1 and quercetin (Tables S5 and S6). Further research is needed to explore whether quercetin regulates the fate of Sertoli cells through these pathways.

## 4. Materials and Methods

### 4.1. Cell Culture and Treatment 

TM4 cells (Mouse Sertoli Cell Line) were purchased from Procell Life Science & Technology Co., Ltd. (CL-0456, Procell, Wuhan, China). The cells were cultured in DMEM/F12 medium (PM150312, Procell, Wuhan, China) supplemented with 10% Horse Serum (164215, Procell, Wuhan, China), 2.5% Fetal Bovine Serum (164210, Procell, Wuhan, China) and 1% Penicillin-streptomycin antibiotics (PB180120, Procell, Wuhan, China). The culture dish was placed in an incubator (Thermo Scientific™ 3111, Waltham, MA, USA) at 37 °C with 5% CO_2_.

Fumonitoxin FB1 (F135527, Aladdin, Shanghai, China) and quercetin (Q111274, Aladdin, Shanghai, China) were dissolved in DMSO before the treatment. TM4 cells were passaged and cultured in 24-well plates. Upon reaching 60–80% confluence, the arrangement of cells became relatively tight, forming one or more layers that were tightly attached to the bottom of the dish, and the cells mainly exhibited spindle and triangular shapes. Then the cells were exposed to medium containing different concentrations of FB1 (0, 20, 40, and 80 μM) or quercetin (0, 5, 10, 20, 40, 60, 80, and 100 μM). Cells cultured in medium containing 0.1% DMSO were designated as the Control group, and all cells were subsequently cultured at 37 °C with 5% CO_2_ for 48 h before additional experiments were conducted.

### 4.2. CCK-8 Assay

TM4 cells were seeded into 96-well plates at a density of approximately 1 × 10^4^ cells per well. Subsequently, cell proliferation rate was assessed using a Cell Counting Kit-8 (CCK-8) Kit (HY-K0301, MCE, Shanghai, China) following the manufacturer’s instruction. CCK-8 utilizes a water-soluble tetrazolium salt, WST-8 (2-(2-methoxy-4-nitrophenyl)-3-(4-nitrophenyl)-5-(2,4-disulfophenyl)-2H-tetrazolium), to evaluate cell proliferation. The WST-8 salt is reduced to a water-soluble, orange-colored formazan dye, with the quantity of formazan dye produced is directly proportional to the number of viable cells. The optical density (OD) value at 450 nm was determined using a spectrophotometer (1530-00183, Thermo Scientific™, Waltham, MA, USA).

### 4.3. TUNEL Assay

Cells were seeded onto 24-well plates containing slides coated with polylysine. Subsequently, the cells were exposed to FB1 or quercetin for a duration of 48 h, and the slides were then fixed in 1 mL of 4% paraformaldehyde for 20 min and rinsed thrice with PBS. Following this, the cells on the slides were subjected to treatment with 1% Triton X-100 for 5 min. TUNEL assay was conducted utilizing a commercially available assay kit (KGA1406, Jiangsu KeyGEN BioTECH Corp., Ltd., Nanjing, China) in accordance with the manufacturer’s guidelines. Fluorescence was detected on a fluorescence microscope (Zeiss, Oberkochen, Germany), and the fluorescence intensity was quantified utilizing ImageJ software (v1.8.0, National Institutes of Health, Bethesda, Rockville, Maryland, MD, USA).

### 4.4. Measurement of ROS, MDA and SOD Activity

The levels of ROS were evaluated utilizing a Reactive Oxygen Species (ROS) Assay Kit (s0033s, Beyotime, Shanghai, China). In brief, cells were cultured in 24-well plates and incubated in medium containing 10 μM DCFH-DA probe at 37 °C for 20 min followed by staining with Hoechst 33258 (C1011, Beyotime, Shanghai, China), and fluorescence was observed using a fluorescence microscope (Zeiss, Oberkochen, Germany). The fluorescence intensity was quantified using a Microplate Reader (SpectraMax iD5, Molecular Devices, San Jose, CA, USA).

For MDA detection, the cells were seeded into the 6-well plate at a density of 5 × 10^5^ cells/mL. Following treatment with FB1 and quercetin, the original medium was aspirated, and the cells were detached by scraping and then centrifuged at 1000 rpm for 10 min. The supernatant was subsequently removed, and a small volume of PBS was added to the cell pellet. The cells were disrupted using an ultrasonic cell crusher (JY92-IIN, SCIENTZ, Ningbo, China) in an ice water bath. The MDA content was quantified utilizing a Malondialdehyde (MDA) assay kit (A003, Nanjing Jiancheng Bioengineering Institute, Nanjing, China), while the activity of SOD was assessed with a Superoxide dismutase (SOD) assay kit (A001-2, Nanjing Jiancheng Bioengineering Institute, Nanjing, China) following the manufacturers’ protocols.

### 4.5. RNA Extraction, cDNA Synthesis, and Real-Time PCR

Total RNAs were extracted from TM4 cells (5 × 10^5^ to 1 × 10^6^ cells per sample) using the TRIzol reagent (15596026, Invitrogen, Carlsbad, CA, USA). RNA purity was assessed with an ultraviolet-visible photometer (BioSpec-nano, Shimadzu, Kyoto, Japan). Subsequently, cDNA was synthesized with the PrimeScript™ RT Reagent kit (RR037A, TaKaR, Tokyo, Japan) following the manufacturer’s protocol. Real-time PCR was conducted as previously described [54]. *Gapdh* was set as the reference gene, and the fold change in gene expression was quantified using the comparative C_T_ method. The primers utilized are detailed in Table S1.

### 4.6. Western Blot 

The TM4 cells, cultured in 6-well plates with approximately 2 × 10^6^ cells per well, were lysed using a buffer (100 mM Tris/HCl pH 7.4, 3% SDS, 10 mM DTT, 17.3% glycerin, and 0.15% bromophenol blue), supplemented with cOmpleteTM Protease Inhibitor Cocktail (04693116001, Roche, Basel, Switzerland). Western blot analysis was conducted following previously described methods [55]. Immunoblotting was carried out using rabbit primary antibodies against SOD1 (1:1000, 10269-1-AP, Proteintech, Chicago, IL, USA), PRDX1 (1:1000, 15816-1-AP, Proteintech, Chicago, IL, USA), PKM2 (1:500, bs-0101R, Bioss, Beijing, China), and LDHA (1:500, 19987-1-AP, Proteintech, Chicago, IL, USA). GAPDH (1:1000, GB15002, Servicebio, Wuhan, China) was utilized as an internal control. The gray values of the bands were quantified using TANON GIS software (TANON, Shanghai, China). 

### 4.7. MMP Detection 

The cultured cells were added to 24-well plates with slides that were coated with polylysine and treated with a Mitochondrial Membrane Potential Test Kit (JC-1) according to the manufacturer’s instructions (C2006, Beyotime, Shanghai, China). Briefly, the slides were incubated with a fluorescence probe JC-1 at 37 °C for 20 min. After incubation, the supernatant was removed and washed with diluted JC-1 dyeing buffer (1x) twice. The fluorescence was detected using a fluorescence microscope (Zeiss, Oberkochen Germany), with excitation at 520 nm and emission at 595 nm for JC-1 aggregates, and excitation at 490 nm and emission at 530 nm for monomers. The fluorescence intensity was quantified using a Microplate Reader (SpectraMax iD5, Molecular Devices, San Jose, CA, USA), and the mitochondrial membrane potential was determined by calculating the ratio of fluorescence intensity of JC-1 in the aggregates state (red fluorescence) to the monomer state (green fluorescence).

### 4.8. Measurement of ATP

The medium containing cells was centrifuged at 4000 rpm for 10 min and the precipitated cells were collected (approximately 10^6^ cells/mL). Subsequently, 300–500 μL of cold ddH_2_O was added to the cells, which were then homogenized in an ice water bath. The cell suspension was further heated in a boiling water bath for 10 min. The ATP content was measured and calculated using an ATP assay kit (A095, Nanjing Jiancheng, Nanjing, China) following the manufacturer’s instructions. 

### 4.9. Determination of Glucose, Pyruvate, and Lactic Acid Levels

The cell suspension was prepared and subsequently centrifuged at 1000 rpm for 10 min. The supernatant was then discarded, and the precipitate was washed twice with PBS. Following another centrifugation at 1000 rpm for 10 min, the cells were resuspended in PBS and disrupted using an ultrasonic cell crusher (JY92-IIN, SCIENTZ, Ningbo, China) in an ice water bath. The glucose content was quantified using a glucose assay kit (A154-1-1, Nanjing Jiancheng, Nanjing, China) in accordance with the manufacturer’s instructions. For the detection of pyruvate and lactic acid, the cell lysates were centrifuged at 2500 rpm for 10 min and the resulting supernatants were collected. The pyruvate content was determined using a Pyruvate test kit (A081-1-1, Nanjing Jiancheng, Nanjing, China), while the lactic acid content was assessed with a Lactic Acid test kit (A019-2-1, Nanjing Jiancheng, Nanjing, China), following the respective manufacturer’s instructions.

### 4.10. Measurement of PKM and LDHA Activity

The medium-containing cells was centrifuged at 4000 rpm for 10 min and the precipitated cells were collected at a concentration of approximately 10^6^ cells/mL. Subsequently, 150–200 μL of pre-cooled PBS was added to resuspend the cell precipitates, and the cells were disrupted using an ultrasonic crusher (JY92-IIN, SCIENTZ, Ningbo, China) in an ice water bath. Following this step, the measurements and calculations of PKM and LDH activities were carried out using a PKM activity assay kit (A076-1-1, Nanjing Jiancheng, Nanjing, China) and an LDH activity assay kit (A020-2, Nanjing Jiancheng, Nanjing, China), respectively, following the manufacturer’s instructions. 

### 4.11. Mitochondrial Staining of Cultured Cells 

The cultured cells were seeded onto 24-well plates containing slides coated with polylysine. Subsequently, they were treated with 500 nM of MitoTracker^®^ Red CMXRos (#9082, Cell Signaling Technology, Boston, MA, USA) and incubated for 30 min at 37 °C. After incubation, the cells were fixed in ice-cold methanol in an ice water bath for 15 min and washed thrice with PBS. Finally, the cells were stained with DAPI and imaged using a fluorescence microscope (Zeiss, Oberkochen, Germany).

### 4.12. RNA-Seq and Transcriptome Data Analysis

Total mRNA was extracted from TM4 cells of the Control, FB1, and FB1 + QR groups, each consisting of three biological replicates. RNA-seq analysis was conducted by Novogene Co., Ltd. Differential expression analysis was carried out using the edgeR R package (version 3.22.5). The *p* values were adjusted using the Benjamini and Hochberg method. A corrected *p*-value of 0.05 and an absolute fold change of 2 were set as the thresholds for determining significantly differential expression. Kyoto Encyclopedia of Genes and Genomes (KEGG) enrichment analysis of the differentially expressed genes was performed using the clusterProfiler R package, and KEGG terms with corrected *p* values less than 0.05 were considered significantly enriched by the differentially expressed genes. The processed RNA-seq data can be found in Tables S2–S6, and the complete dataset has been deposited in the NCBI Sequence Read Archive (SRA) database.

### 4.13. Statistical Analysis

Statistical comparisons were performed using the Student’s unpaired *t*-test or one-way ANOVA (with Tukey’s multiple comparisons test as the post hoc test). All experiments were performed in triplicate, and results were presented as mean ± standard error of the mean (SEM). Statistical significance was set at *p* < 0.05.

## 5. Conclusions

In conclusion, this study proves that quercetin effectively restores impaired cell proliferation and function in Sertoli cells treated with FB1. This restoration is attributed, in part, to the reduction of excessive ROS production and enhancement of the glycolysis process. The findings presented in this study elucidate the protective mechanisms of quercetin against male reproductive toxicity induced by FB1.

## Data Availability

The raw data of RNA-Seq have been deposited in the NCBI Sequence Read Archive (SRA) database with the accession number SRR29790688-SRR29790696. Other data presented in this study are included in the published article and Supplementary Materials. Further inquiries can be directed to the corresponding author.

## Supplementary Materials

The following supporting information can be downloaded at: https://www.mdpi.com/article/10.3390/ijms25168764/s1.
