# Supplementary figures and images for: The Protective Effect of Quercetin against the Cytotoxicity Induced by Fumonisin B1 in Sertoli Cells
Source: Int J Mol Sci. 2024 Aug 12;25(16):8764. doi: 10.3390/ijms25168764 (PMC11355056; doi:10.3390/ijms25168764)

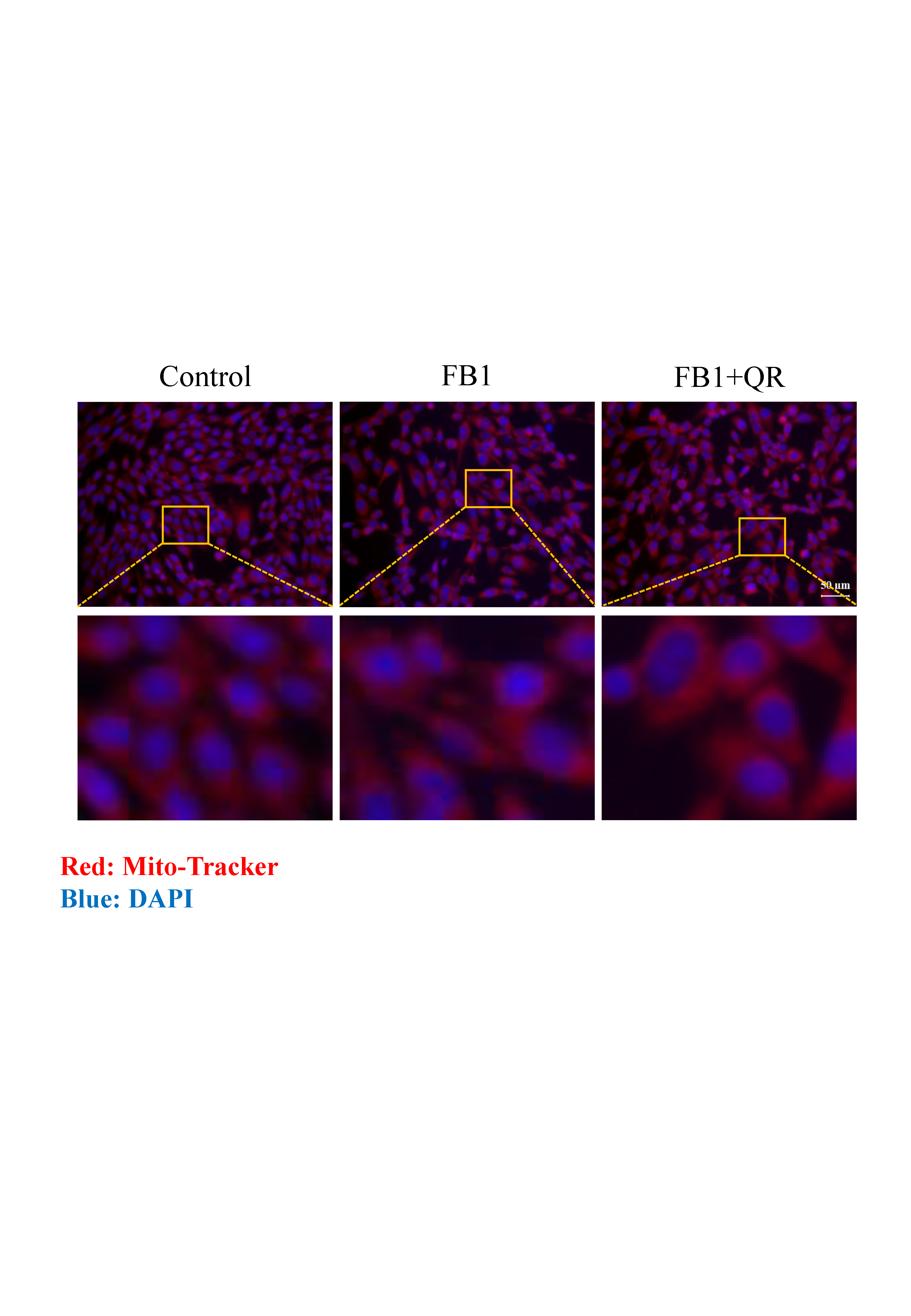

Supplement: Supplementary file 1 [file ijms-25-08764-s001.zip › Fig S1.tiff]

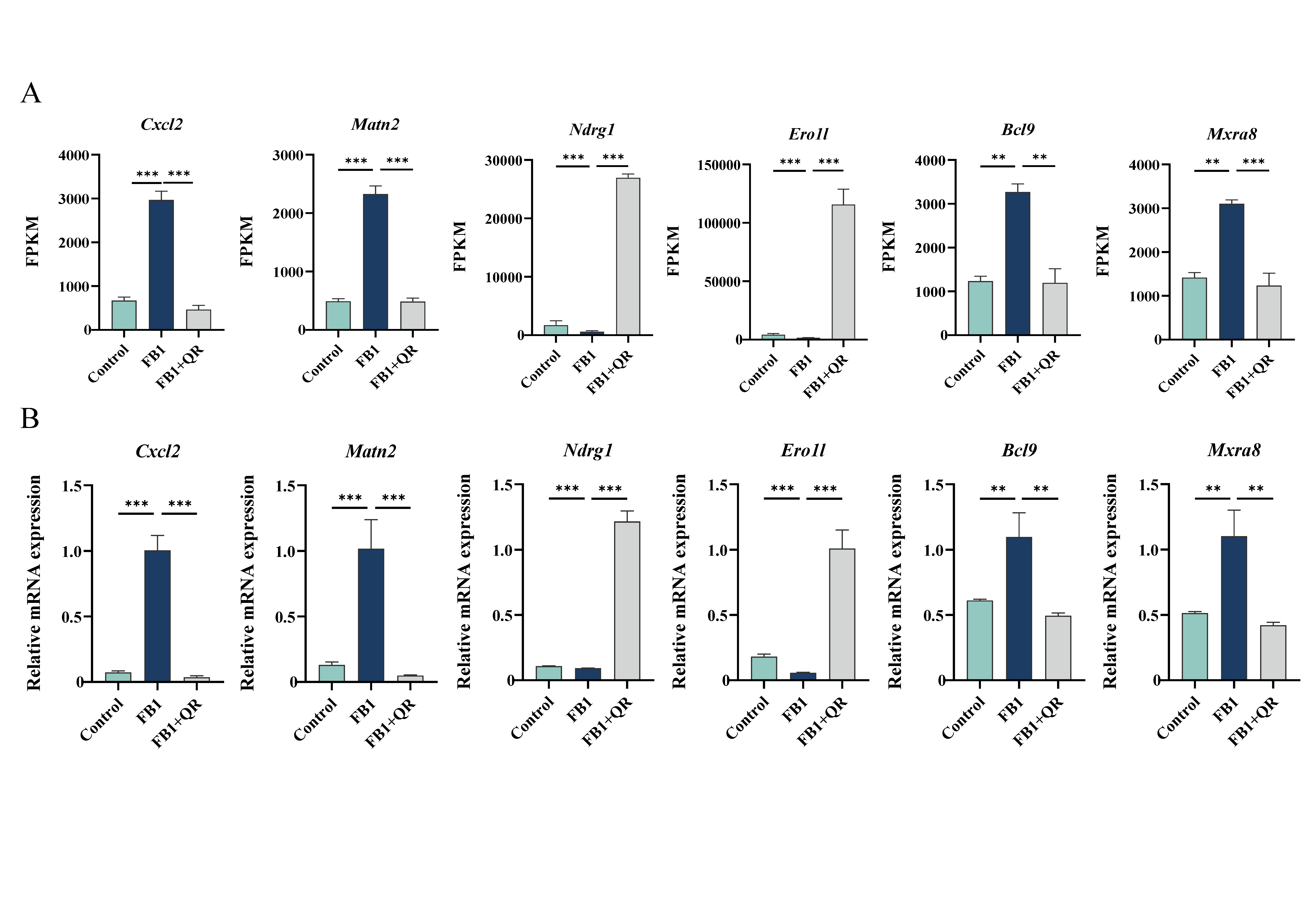

Supplement: Supplementary file 1 [file ijms-25-08764-s001.zip › Fig S2.tiff]

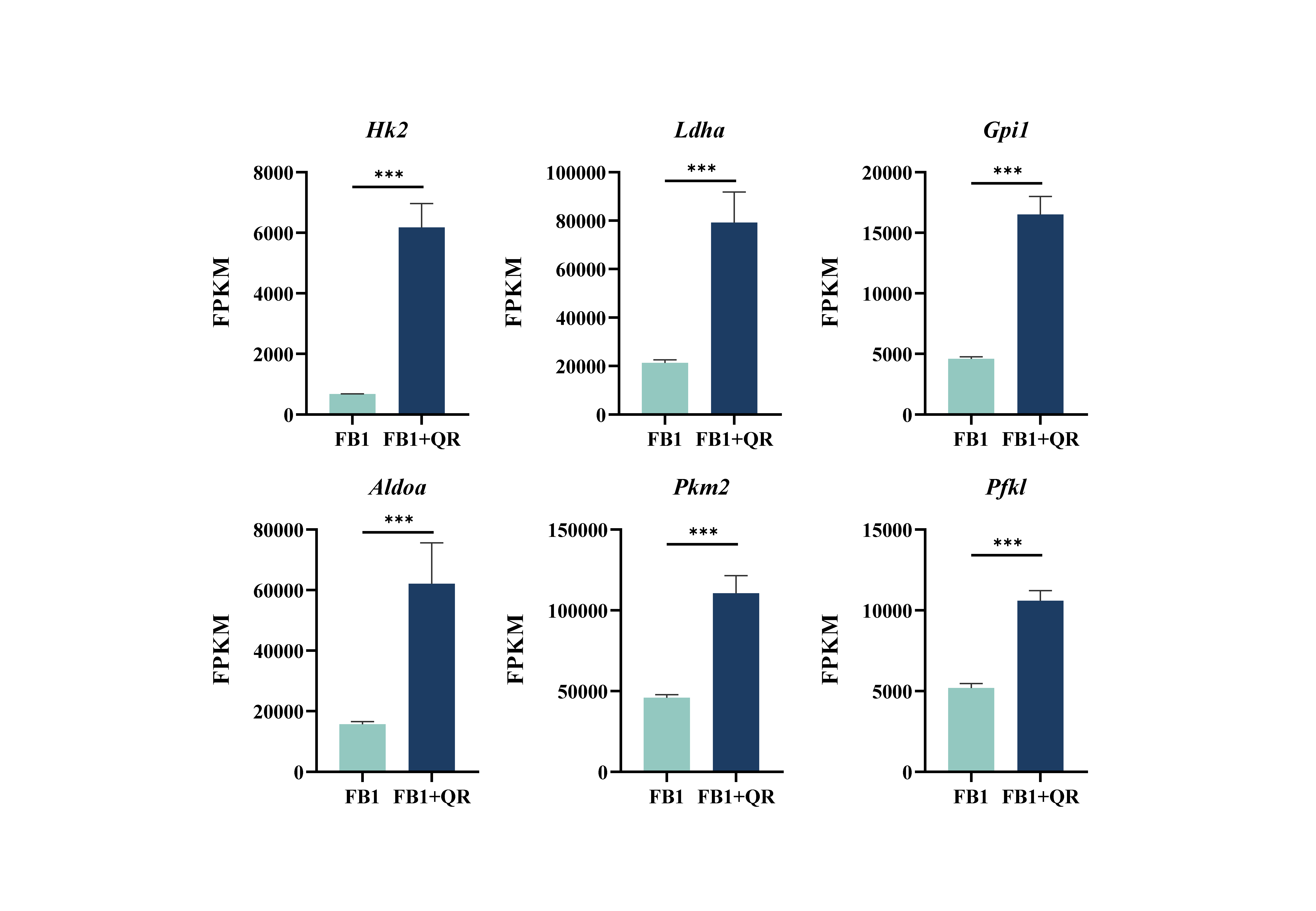

Supplement: Supplementary file 1 [file ijms-25-08764-s001.zip › Fig S3.tiff]
